# The Use of Platelet-Rich Plasma in De Quervain’s Tenosynovitis: A Systematic Review

**DOI:** 10.7759/cureus.74232

**Published:** 2024-11-22

**Authors:** Mahmood Alam, Ayman Merza Abdulla Mohamed, Mahmood Alawainati, Fayza Haider

**Affiliations:** 1 Orthopedics and Trauma, Salmaniya Medical Complex, Manama, BHR; 2 Orthopedics and Trauma, American Mission Hospital, Manama, BHR; 3 Medicine, Royal College of Surgeons in Ireland, Manama, BHR; 4 Family Medicine, Primary Healthcare Centers, Manama, BHR; 5 Pediatric Surgery, Salmaniya Medical Complex, Manama, BHR; 6 College of Medicine and Medical Sciences, Arabian Gulf University, Manama, BHR

**Keywords:** de quervain disease, de quervain tenosynovitis, first extensor compartment, platelet-rich plasma (prp), radial wrist pain

## Abstract

De Quervain's disease (DQVD) is the stenosing tenosynovitis of tendons in the first extensor compartment of the forearm. It is a common inflammatory condition that is often treated conservatively. While conservative therapy is an effective modality of treatment for a large number of patients suffering from DQVD, some patients do not improve with conservative measures. Many alternative treatment modalities are recognized in the treatment of DQVD. One of the non-surgical treatment strategies that is currently on the rise is platelet-rich plasma (PRP) injections into the first dorsal extensor compartment. It is thought that PRP injections contain growth factors that may provide a regenerative stimulus to tendon healing. Various studies evaluated as a treatment modality for different tendinopathies including DQVD. There remains, however, controversy as to the efficacy of its benefit and efficacy in treating DQVD.

This paper is a systematic review of the literature conducted to evaluate the effectiveness of PRP in the treatment of DQVD. The review was conducted in line with PRISMA guidelines for systematic reviews. The review included a systematic search through PubMed, Embase, Cochrane Library, Medline, Amed, and the Web of Sciences, and was supplemented by manual search through other published online resources. The period of search was defined as January 2013 to October 2023, and the search included all studies that evaluated the use of PRP in the treatment of DQVD. A total of 1,029 records were screened; only eight studies met the inclusion criteria and were included in the study.

Three randomized clinical trials and five experimental studies were included in the review. A systematic review of the evidence suggested that PRP is a promising and safe alternative to conventional steroid injections in the treatment of De Quervain’s tenosynovitis. Further large sample studies are needed prior to the definitive recommendation of PRP as the gold standard for the treatment of De Quervain’s tenosynovitis.

## Introduction and background

De Quervain's disease (DQVD) is the stenosing tenosynovitis of tendons in the first extensor compartment of the forearm. While several theories exist about the etiology of the condition, a predominant hypothesis is that it is the result of repetitive microtrauma caused by monotonous gliding of the tendons of the first extensor compartment (abductor policis longus and extensor policis brevis) over the radial styloid causing thickening and inflammation of the extensor wrist retinaculum [1,2]. The predominant view in the literature is that tendon microtrauma can lead to individual tendon fibril degeneration due to stress across the tendon; this stress accumulates over time leading to chronic degeneration and tendinopathy [3]. The condition is common in pregnant and lactating women and shows increased prevalence in the older age groups (fifth and sixth decades of life) [4]. The diagnosis of DQVD is usually clinical, often utilizing the well-established Finkelstein test [2]. Other modalities have been utilized to help confirm the diagnosis and measure treatment response such as magnetic resonance imaging, computed tomography, and ultrasound imaging. Chief among those modalities is the musculoskeletal ultrasound (MSUS), which has been utilized not only to diagnose and detect responses to treatment but also to guide the accurate administration of injectable medications into the first extensor compartment [5].

Many treatment modalities are recognized in the treatment of DQVD; the first line of treatment is usually conservative. Patients usually undergo a period of activity modifications, splinting, occupational or physical therapy and they are often prescribed non-steroidal anti-inflammatory medications [6]. Conservative therapy is an effective modality of treatment for a large number of patients suffering from DQVD [7]. Authors have, however, suggested in a systematic review that combining conservative management with intralesional injection of steroids resulted in better outcomes than used alone [8]. Steroid injections are widely used injectable drugs for the treatment of DQVD; with studies citing up to 83% pain relief with a single corticosteroid injection [9,10]. However, local corticosteroid injection can carry the risk of local skin complications in up to 20% of cases, including depigmentation of the skin, skin atrophy, and thinning [11]. Furthermore, a recurrence rate of up to 32%-48% within a year has been reported after treatment of DQVD with corticosteroid alone [11,12].

Patients who are refractory to conservative treatment and local steroid injections have been traditionally offered surgical management in the form of first dorsal extensor compartment release. Surgery is, however, not without its drawbacks. Several complications have been reported with the surgical release including the incomplete release of the compartment with retained sub sheaths, injury to the superficial branch of the radial nerve, formation of a painful neuroma, hypertrophic scarring, and tendon subluxation [13].

The invasive nature of surgery coupled with the undesirable side effect profile of and relatively high recurrence rates of DQVD managed by corticosteroid injection has led to a rise in interest in alternative treatment modalities. One of the non-surgical strategies of treatment that is currently on the rise is platelet-rich plasma (PRP) injections into the first dorsal extensor compartment. It is thought that PRP injections contain growth factors that may provide a regenerative stimulus to tendon healing. The presence of PRP in an area of inflammation or degeneration enhances the natural processes of healing by bringing molecules that accelerate functional recovery and regenerate tissue into the sites of injury or inflammation [14-16]. These regenerative properties created interest in their use to treat degenerative tendinopathies including DQVD.

Various studies have shown PRP to be a safe and effective treatment modality for different tendinopathies including patellar, rotator cuff, and Achilles tendinopathies [17-22]. The efficacy of PRP in the treatment of various tendinopathies sparked interest in its use for the treatment of DQVD.

Several authors evaluated the efficacy of PRP in the treatment of DQVD with most of the studies finding PRP as an effective and safe alternative to other injections [23-30]. While the evidence for the efficacy of PRP in DQVD is growing, most of the studies conducted had relatively small sample sizes with findings that are not easily generalizable to a broad range of populations. A systematic review is essential to compile the findings of such studies and to provide a general view of the strength of the current evidence for the use of PRP in DQVD and to identify areas of growth that further studies could address in order to provide robust evidence supporting the use of PRP in DQVD.

While several scoring schemes have been devised to measure the response to therapy of wrist and hand conditions; most studies have adopted the use of the visual analog score (VAS) and the disabilities of the arm, shoulder, and hand score to measure the efficacy of interventions in the treatment of DQVD [23-28,30]. The simplicity, reproducibility, and widespread use of these scores have led to their adoption in most of the studies that attempt to conclude the efficacy of various treatment modalities around the wrist and hand region. This systematic review aims to evaluate the efficacy of PRP injection to the first dorsal extensor compartment in the treatment of DQVD.

## Review

Methods 

The study was designed in line with the current PRISMA guidelines for systematic reviews. Available Online databases listed in the methods section were searched for all available publications pertaining to the use of PRP in the treatment of De Quervain’s tenosynovitis. The quality of evidence was then evaluated using the Joana Briggs Institute (JBI) assessment tool, and an analysis was conducted of the relevance of each study to clinical practice. The quality of studies to be included in the systematic review was assessed independently by two of the authors, the study was only included if both authors agreed it met the standards of the Joana Briggs Institute assessment and was of relevance to the use of PRP in the treatment of DQVD. Where disagreement was present in the assessment of study quality, the most senior author of the project was invited as a third assessor to independently assess the study in question. The assessment of the third assessor will be the final determining factor of inclusion or exclusion of a particular study in the case of a tie between the first two assessors. The studies were then reviewed with an assessment of the effect of the use of PRP on various outcome measures including pain scores, functional scores, ultrasonographic effect of injection, and other parameters when available.

The study was designed as a systematic review. The study was conducted in the year 2023 and the search period included studies published in the period between January 2013 and October 2023.

Identifying relevant studies

Search Strategy

A systematic literature search was part of the capstone project. The search involved PubMed, Embase, Cochrane Library, Medline, Amed, and the Web of Sciences. In addition, the authors conducted a manual online search of published literature to ensure the inclusion of any relevant study that may not be indexed in the databases searched. The references of the selected papers were then screened for eligibility.

The search terms were De Quervain tenosynovitis OR de Quervain’s disease OR Repetitive strain injury OR Abductor pollicis longus OR Extensor pollicis brevis OR first extensor compartment tenosynovitis OR de Quervain tendinopathy AND platelet enriched plasma OR wrist injection OR injection OR PRP OR platelet-rich plasma.

Study Selection

All the retrieved results of the seven resources were exported to an Excel file and organized according to the title of the study, author, and journal. After that, duplicated studies were removed from the list and a new list of the studies was made. Then, the electronic databases were assessed for appropriateness based on the title of the studies and the details of the abstracts. After this, a new final list of eligible studies was formulated.

Studies that were not written in English, studies with irrelevant outcomes, clinical reports, case series, and studies with unavailable full texts will be excluded. The details of the selection criterion are presented in Table 1. Duplicates were removed. Then, the studies were assessed and screened for appropriateness; specifically, the studies’ titles and abstracts were assessed for eligibility and appropriateness. The final step involved assessing the full-text articles for eligibility and appropriateness.

**Table 1:** Selection criteria of the systematic review PRP - platelet-rich plasma, DQVD - De Quervain's disease, DASH - Disabilities of the Arm, Shoulder, and Hand, VAS - Visual analog scoring

Inclusion criteria	Exclusion criteria
Use of PRP in the treatment of DQVD and use of quantifiable outcome measures such as ultrasound tendon thickness, DASH, or VAS scoring (or equivalent) to detect treatment efficacy	Non-English written manuscript
	Unavailability of the full-text manuscript
	Review articles
	Case reports and case series
	Poor quality studies as detailed in the quality assessment section

**Figure 1:**
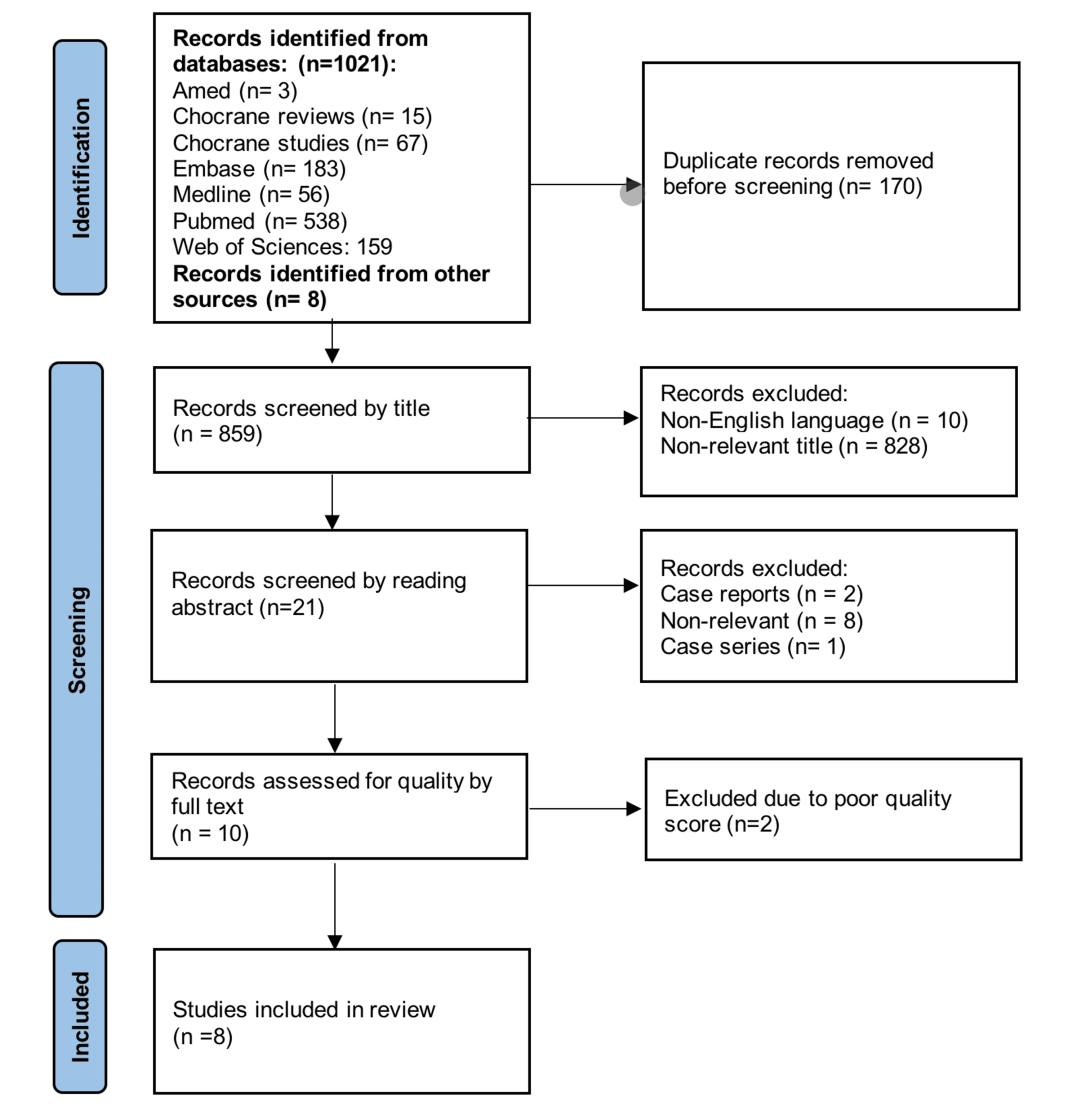
PRISMA flowchart

A total of 1,021 records were retrieved through database search, and eight records were identified through manual search. Records were then screened by title and abstract for relevance. Ten studies were identified as relevant and were included in the quality assessment for inclusion in this systematic review. Two studies were excluded due to poor quality assessment scores leaving eight studies that were included in the review. A flowchart breakdown of the studies identified is included in Figure 1.

Ethical considerations

Ethical approval was obtained from the local authority in the governmental hospitals in the Kingdom of Bahrain. 

Quality assessment

The shortlisted studies were independently assessed by two of the authors using the JBI bias assessment tools. The studies were first classified according to their protocol as either randomized clinical trials (RCT) or experimental studies with or without randomization. Two tools were used in the quality assessment depending on the type of study in question. The RCT tool was used for three studies and the experimental study tool was used for seven studies. The tools utilized assessed the study in question utilizing a checklist format to determine the risk of bias in each of the studies in question.

Each study was given a numerical JBI score according to the tool used, and each assessor outlined a final decision to include or exclude each of the assessed studies with justification for exclusion if applicable. Each of the assessors was blinded to the assessment of the other author to minimize the risk of bias. Of the 10 studies that were included in the quality assessment, both assessors agreed to include seven studies. One study was excluded by both authors due to poor quality; two studies were the subject of a tie with one of the assessors including it and another excluding it. The tie was broken according to the protocol by invitation of the third assessor who decided to include one of the studies and exclude the other one. A summary table of the characteristics of the included studies with quality assessment of both independent assessors is included in Table 2.

**Table 2:** Quality assessment and characteristics of included studies JBI = Joana Briggs Institute score, PRP = Platelet-rich plasma, n = Number, Vs = Versus, wk = Weeks, mo = Months, mg = Milligrams

Number	Study, year	Country	Period	Study design	Mean JBI	Sample size	Randomization	Demographics	Comparison or control group	Dose & frequency of PRP	follow up
1	El sheik et al., 2020 [23]	Egypt	2020	Prospective experimental comparative study	7.5/9	n = 40	Not randomized	Males=4, Females=31	corticosteroid comparison group	1mL of betamethasone injected once Vs 1.5mL of PRP, injected once.	Pre injection, 1mo, 6mo
2	Kumar et al., 2023 [24]	India	2022	Prospective randomized rxperimental comparative study	8/9	n = 60	Randomized	Unclear	corticosteroid comparison group	3mL of PRP injected once Vs 1mL of methylprednisolone injected once.	Pre injection, 1mo, 3mo, 6mo, 1 year
3	Gulati et al., 2022 [25]	India	September 2020 to September 2022	Prospective randomized experimental comparative study	8/9	n = 40	Randomized	Males =21, Females = 13 Right hand = 29, Left hand = 15	corticosteroid comparison group	3-4mL of PRP injected 3 times (0,4wk, 8wk) Vs 40mg of triamcinolone injected 3 times (0,4wk, 8wk).	Pre injection, 4wk, 12thwk, 24thwk.
4	Giroti et al., 2021 [26]	India	February 2018 to June 2019	Prospective experimental comparative study	6.5/9	n = 50	Not randomized	Males = 5, Females = 42	corticosteroid comparison group	1mL of methylprednisolone with 1mL of 0.5% Bupivacaine injected once Vs 3-4mL of PRP, injected once.	Pre injection, 1mo, 6mo
5	Shoma et al., 2023 [27]	Bangladesh	2022	Randomized clinical trial	7/13	n = 100	Randomized	Males = 28, Females = 66 Right hand = 62, Left hand = 32R	PRP vs corticosteroids vs conservative management	3mL of PRP injected once, 40mg of triamcinolone injected once	Pre injection, 1mo, 3mo, 6mo
6	Rahman et al., 2023 [28]	Bangladesh	March 2020 to February 2021	Randomized clinical trial	6/13	n = 54	Randomized	Males = 9, Females = 42, Right hand = 23, Left hand = 18	PRP vs conservative management	3mL of PRP injected once	Pre injection, 1mo, 3mo, 6mo
7	Lu et al., 2017 [29]	China	July 2014 to March 2015	Randomized Controlled trial	7.5/13	n = 52	Randomized	Males = 14 Females = 37	surgery alone Vs Surgery and PRP	3mL of PRP injected once with surgery	Pre injection, 3mo, 6mo, 12mo
8	Asaad et al., 2023 [30]	Iraq	January 2020 to February 2021	Prospective experimental study	5/9	n = 12	Not randomized	Males = 0, Females = 12 8 affecting dominant hand, 4 affecting non dominant hand	no control or comparison group	2mL of PRP, injected once.	Pre injection, 1wk, 1mo, 3mo

Results

Overview of the Studies Included

This systematic review included a total of eight studies, three of which were randomized controlled trials and five of which were experimental. The studies were conducted in the period between 2017 and 2023. Four of the studies compared the use of PRP against the use of conventional injection therapy in the form of corticosteroids, one study compared PRP versus steroids or conservative management, one study compared PRP to conservative management alone, one study compared surgical intervention with PRP injection versus without PRP injection, and one study did not have a comparison group.

Three studies were conducted in India, two were conducted in Bangladesh, one was conducted in China, one was conducted in Egypt, and one was conducted in Iraq. The sample size of the studies was variable; the smallest study Included 12 hands, and the largest study included 100 hands. The mean sample size across all studies was 51 with a standard deviation of 24. The mean age of the participants in the studies ranged from 35.8 to 47.6 years; the overall mean age across all studies was 42.5 years with a standard deviation of 4. All studies in which the gender of subjects was noted showed a female predominance with the exception being the study conducted by Gulati et al. which showed a male predominance in the subject groups.

All studies except Leu et al. [20] used Visual Analogue Scoring (VAS) as the main outcome measure, four studies used the Disabilities of the Arm, Shoulder, and Hand score (DASH) as a second outcome measure, two studies used Patient Rated Wrist Evaluation score (PRWE) as a second outcome measure, and two studies used the Mayo wrist score as a second outcome measure. Two studies measured radiological outcomes in the form of ultrasound assessment of the thickness of the tendon sheath, and extensor retinaculum.

All studies included patients based on a clinical diagnosis of De Quervain’s tenosynovitis; while some variability in inclusion criteria was noted, most of the studies had similar inclusion and exclusion criteria overall. Most studies excluded patients with rheumatoid arthritis, and patients who received steroid injections previously or have recently used non-steroidal anti-inflammatory drugs. A summary of various scores is detailed in Tables 3, 4.

**Table 3:** Summary of VAS and DASH scores of studies included in the review VAS = Visual Analogue score, DASH = Disabilities of the Arm, Shoulder, and Hand score, TT = Tendon thickness, PRP = Platelet-rich plasma, mo = Months, wks = Weeks

	Study, year	Study design	VAS score		DASH score		Ultrasound findings		Complications
1	El Sheik et al., 2020 [23]	Prospective experimental study	PRP group	Steroid group	PRP group	Steroid group	PRP group	Steroid group	
			Baseline = 7.70±1.95	Baseline = 7.65±1.93	Baseline = 36.45±8.67	Baseline = 34.52±8.13	Baseline TT = 2.55±0.62	Baseline TT = 3.1±1.02	
			1mo =2.65±2.98, P=0.005	1mo = 0.55±1.05, P=0.005	1mo =11.13±13.04, P=0.043	1mo = 5.34±3.48, P=0.043	1mo =2.14±0.8, P=0.857	1mo = 2.18±0.58, P=0.857	
			6mo =1.94±3.04, P=0.034	6mo = 2.13±2.75, P=0.034	6mo =9.38±13.52, P=0.729	6mo = 10.9±10.86, P=0.729	6mo =2.60±0.8, P=0.318	6mo = 2.37±0.59, P=0.318	
2	Kumar et al., 2023 [24]	Prospective randomized experimental study	PRP group	Steroid group	PRP group	Steroid group	-		26% complication rate with steroids P=0.026
			Baseline = 6.73±1.44	Baseline = 6.53±1.48	Baseline = 27.53 ±6.46	Baseline = 26.98±7.02			
			1mo = 3.67±2.6, P=0.795	1mo = 3.27±2.33	1mo = 11.37±9.46, P=0.829	1mo = 10.84±9.41			
			3mo = 1.87±1.78, P=0.787	3mo = 2.3±2.32	3mo = 5.88±6.56, P=0.633	3mo = 6.82±8.7			
			6mo = 0.83±0.99, P=1	6mo = 1.23±1.61	6mo = 2.38±3.87, P=0.587	6mo = 3.02±5.13			
			12mo = 0.40±0.62, P=1	12mo = 0.47±0.78	12mo = 0.49±0.85, P=0.183	12mo = 1.21±2.83			
3	Gulati et al., 2022 [25]	Prospective randomized experimental study	PRP group	Steroid group	PRP group	Steroid group	-		-
			Mean 4wks = 5, P<0.001	Mean 4wks = 7	Mean 4wks = 93.1, P<0.001	Mean 4wks = 61.3			
			Mean 12wks = 3.5, P<0.001	Mean 12wks = 5	Mean 12wks = 87.2, P<0.001	Mean 12wks = 40.9			
			Mean 24wks = 1, P<0.001	Mean 24wks = 5	Mean 24wks = 72.7, P<0.001	Mean 24wks = 13.6			
4	Giroti et al., 2021 [26]	Prospective experimental study	PRP group	Steroid group	PRP group	Steroid group	-		-
			Baseline = 7.5	Baseline = 7.8	Baseline = 7.5	Baseline = 7.8			
			Post injection = 1.4	Post injection = 2.1	Post injection = 1.4	Post injection = 2.1			
5	Shoma et al., 2023 [27]	Randomized clinical trial	PRP group	Steroid group	-		-		Minor bleeding occurred in a single patient in PRP group
			Baseline = 7.8±0.8	Baseline = 7.9±0.6					
			1mo = 5.6±0.7, P=0.097	1mo = 5.3±0.5					
			3mo = 3.5±0.7, P=0.001	3mo = 3.9±0.5					
			6mo = 1.7±0.7, P<0.001	6mo = 2.9±0.6					
6	Rahman et al., 2023 [28]	Randomized clinical trial	PRP group	Conservative therapy group	-		-		11.5% of patient in the PRP group complained of injection site pain
			Baseline = 7.3±0.8	Baseline = 7.1±0.6					
			1mo = 3.4±0.5, P<0.001	1mo = 4.3±1.1					
			3mo = 1.5±0.8, P<0.001	3mo = 3.4±1.0					
			6mo = 1.1±0.3, P<0.001	6mo = 2.0±0.8					
7	Asaad et al., 2023 [30]	Prospective experimental study	Baseline average = 8.66		-		Mean baseline retinaculum thickness = 1.89±0.5		2 vasovagal attacks reported
							Mean retinaculum thickness (1mo) = 1.3±0.6		
			Post treatment (1mo) = 4.5				Mean retinaculum thickness (1mo) = 0.96±056 (P<0.001)		
			Post treatment (3mo) = 1.9						
			(P<0.001)						

**Table 4:** Summary of PRWE scores and Mayo wrist scores of studies included in the review PRWE = Patient-Rated Wrist Evaluation, PRP = Platelet-rich plasma, mo = Months, wks = Weeks

	Study, year	Study design	PRWE score		Mayo Wrist Score		Complications
1	Kumar et al., 2023 [24]	Prospective randomized experimental study	-		PRP group	Steroid group	26% complication rate with steroids P=0.026
					Baseline = 64 ±6.22	Baseline = 64.83±5.65	
					1mo = 75.5±11.32, P=0.954	1mo = 75.67±11.2	
					3mo = 82.83±8.68, P=0.722	3mo = 82±9.34	
					6mo = 88.83±6.91, P=0.274	6mo = 86.83±7.13	
					12mo = 92.5±4.1, P=0.208	12mo = 90.83±5.88	
2	Shoma et al., 2023 [27]	Randomized clinical trial	-		PRP group	Steroid group	Minor bleeding occurred in a single patient in PRP group
					Baseline = 40.7 ±5	Baseline = 39.2±3.7	
					1mo = 66.7±4.8, P<0.001	1mo = 56.8±5.8	
					3mo = 75.3±4.7, P<0.001	3mo =65.8±4.5	
					6mo = 87.9±3.7, P<0.001	6mo = 73.7±4.8	
3	Rahman et al., 2023 [28]	Randomized clinical trial	PRP group	Conservative therapy group			11.5% of patients in the PRP group complained of injection site pain
			Baseline = 75.1±3.8	Baseline = 73.0±4.8			
			1mo = 27±8.3, P=0.021	1mo = 32.8±9.2			
			3mo = 17±2.4, P<0.001	3mo = 27.8±9.2			
			6mo = 8.2±2.9, P<0.001	6mo = 20±5.7			
4	Lu et al., 2017 [29]	Randomized controlled trial	PRP + Surgery	Surgery alone	-		-
			Baseline = 51.58 ±8.903	Baseline = 52.788±6.073			
			3mo = 28.64±4.24, P<0.001	3mo = 23.058±5.156			
			6mo = 14.22±2.479, P<0.001	6mo = 18.135±3.799			
			12mo = 10.52±2.489, P<0.001	12mo = 18.692±3.572			

Quality Assessment of the Studies Included

The quality of the studies was assessed independently by two assessors using the Joana Briggs Institute (JBI) tools for randomized controlled trials and experimental trials. A mean score was calculated from the independent assessment of both assessors. Of the initial 10 studies identified during the literature search, both assessors agreed to include seven. One study was excluded by both assessors due to poor quality, and two papers received a tie (included by one reviewer and excluded by another). Of the two papers that received a tie, one was included after review by a third most senior independent reviewer, and one was excluded. Overall, eight papers were included in the final analysis of this systematic review.

Quality assessment scores (JBI Scores) within the experimental group of studies varied with the highest score of 8/9 achieved by two studies [24,25] and the lowest score being 5/9 [30]. Within the Randomized controlled trails group the lowest score was 6/13 [28] and the highest score was 7.5 [20]. Mean score for the experimental group was 7 with a standard deviation of 1.27, while the mean score for the randomized controlled trial group was 6.83 with a standard deviation of 0.76. Five studies were randomized, but none of the studies were blinded. The randomization procedure was not well detailed in any of the studies.

Studies included a minimum follow-up of three months and a maximum duration of follow-up that extended to one year. All studies evaluated the patients at baseline and subsequently for a minimum of two visits. Four studies reported complete follow-up of all subjects included, the remaining four studies reported minimal attrition with only 16 patients being lost to follow-up across all studies. Loss of follow-up did not significantly impact the individual samples of the studies included in the review.

Procedure and Technique of PRP Preparation and Administration

Of the eight studies included in this analysis, only five reported the details of the preparation and administration of PRP injected. While there was some variation in the dose of PRP administered across the studies, most studies used a similar dose of PRP and used a single injection protocol. Five studies injected 3mL of PRP in a single session, one study injected 2mL of PRP in a single session, and one used 1.5mL of PRP in a single session. Only one study used multiple injections of PRP separated across time [25], the authors injected 3mL of PRP at the initial visit, and subsequently at four weeks and eight weeks.

Preparation of PRP prior to administration was highly variable as was the concentration of platelets in the solution injected. Blood collected by venesection was centrifuged at variable speeds, with the lowest speed of 900 rounds per minute (rpm) and the highest speed registered at 800Hz (48,000 rpm). Six studies conducted a single centrifuge round while two studies centrifuged the collected blood twice. Two studies used ultrasound to guide the injection of PRP into the tendon sheath, and one of those studies also performed a percutaneous tenotomy at the time of injection [30].

Clinical and Radiological Outcomes

Visual analog scores: Seven studies reported the use of VAS analog scores in the assessment of the efficacy of PRP injections [23-28,30]. Six studies showed improvement in VAS scores during follow-up across all follow-up intervals in comparison to baseline scores [23,25-27,30]. The improvement in VAS scores in comparison to baseline scores was statistically significant across all six studies. One study [25] did not measure VAS at baseline.

Five studies compared VAS between patients injected with PRP versus corticosteroids [23-27]. Two studies reported a statistically significant reduction in VAS favoring PRP over steroids across all time points [25,28]. One study [27] showed a faster initial reduction in VAS in the steroid group that was not statistically significant, but more reduction in VAS in the PRP group at three months which reached statistical significance in favor of PRP. One study [23] showed a faster initial reduction in VAS in the steroids group that reached statistical significance, but more reduction in the PRP group at six months. One study [24] showed that both PRP and steroids improved VAS comparably, with no statistically significant difference at various time points.

Two studies compared VAS scores between patients injected with PRP versus conservative management [27,28]. Both studies showed statistically significant improvement in VAS favoring PRP when compared to conservative management alone at all time points.

Disabilities of the Arm, Shoulder, and Hand score: Three studies reported statistically significant improvement in DASH scores after injection of PRP across all time points when compared to baseline DASH scores [23,24,26]. One study [25] did not measure DASH scores at baseline.

Four studies compared DASH scores between patients injected with PRP versus corticosteroids [23-26]. Two studies showed statistically significant improvements in DASH scores favoring PRP over corticosteroids across all time points [25,26]. One study [23] showed a statistically significant improvement in DASH scores favoring steroids at one month, while DASH scores improved more with PRP at six months, but this improvement was not statistically significant when compared to steroids. One study [24] showed that both PRP and steroids improved DASH comparably, with no statistically significant difference at various time points.

Patient-rated wrist evaluation score (PRWE): Two studies reported the use of PRWE analog scores in the assessment of the efficacy of PRP injections and surgery for the treatment of De Quervain’s tenosynovitis [28,29]. Lu et al. reported improvement of PRWE scores across all time points compared to baseline with surgery alone and with surgery combined with PRP injection. The addition of PRP injection to surgical intervention resulted in a statistically significant improvement of PRWE scores at six and 12 months in comparison to surgery alone, while surgery alone resulted in a more significant improvement of PRWE scores at three months.

Rehman et al. reported improvement in PRWE scores with PRP injection across all time points when compared to baseline. He also reported statistically significant improvement of PRWE scores with PRP over conservative management across all time points.

Mayo wrist score: Two studies reported statistically significant improvement in Mayo wrist scores after injection of PRP across all time points when compared to baseline scores [24,27]. Shoma et al. reported statistically significant improvement of mayo wrist scores favoring PRP over corticosteroids and conservative management across all time points. Kumar et al., however, showed no statistically significant improvement in Mayo scores between the PRP and steroid groups.

Radiological Outcomes

Two studies assessed the change of sonographic parameters in response to PRP injection [23,30]. El Shiekh et al. reported a reduction in tendon thickness, tendon sheath thickness, and extensor retinaculum thickness at one and six months of baseline. There was, however, no statistically significant difference in the reduction of all three parameters when comparing steroids to PRP at one month. However, a significant reduction in extensor retinaculum thickness was reported at six months favoring the PRP group. Assad et al. reported a statistically significant reduction in tendon sheath effusion, retinaculum thickness, and peritendinous hyperemia at various follow-up points in comparison to baseline.

Complication Profile

Six studies commented on the complications during the conduction of their protocol [24,26-30]. Two studies did not mention if any complications occurred [23,25]. Overall, PRP injection resulted in three cases of minor local pain that resolved with ice application. Two cases of vasovagal attacks, and two cases of minor bleeding. Steroid injection on the other hand resulted in four cases of depigmentation, and two cases of recurrence of symptoms after initial improvement.

Discussion

Various modalities have been used in the treatment of tendinopathies across the years; while conservative management of tendinopathies in the form of splinting and NSAID administration remains the mainstay first-line treatment, recent advancement in treatment promise faster pain relief and earlier recovery of function [31,32]. Peritendinous steroid injections have been traditionally used as a therapeutic option for patients who suffer from chronic tendinopathy that is refractory to conservative management [10,33]. While various studies have proven the effectiveness of steroid injections in managing tendinopathy, their use has been associated with some complications including skin atrophy, depigmentation, and tendon rupture [34-36]. Recent studies focused on finding alternative modalities of treatment that promise similar or superior efficacy to that of steroid injection while enjoying a safer side effect profile. PRP has emerged as a promising alternative to steroid injections in the treatment of various tendinopathies [37].

PRP is an autologous blood product that concentrates a large number of platelets in a small volume of plasma [16,38,39]. It has been hypothesized to accelerate the body’s own healing process by releasing growth factors that stimulate stem cells to produce new host tissue [16]. This healing potential has raised prospects of the use of PRP in various medical disciplines including orthopedic surgery, plastic surgery, dentistry, and even cardiac surgery [40-42]. The usefulness of PRP has gained traction in various fields primarily due to promising results of efficacy coupled with the great safety profile and relatively few contraindications for its use [16].

While tauopathies are a heterogeneous group of conditions, they are often treated similarly with both local and systemic interventions [43,44]. PRP is a novel treatment that is on the rise to address various forms of tendinopathies [21,45,46]. Various studies have explored the use of PRP in Achilles tendinopathy [19,20], rotator cuff tendinopathy [17,18], and patellar tendinopathy [22], with varying degrees of success. Most of the studies have found encouraging preliminary results that need further scrutiny with large-scale trials to clarify the efficacy of PRP in the treatment of various types of tendinopathies. The potential of PRP in the treatment of these chronic conditions may offer a safer alternative to the conventional use of steroid injections that may be associated with undesirable side effects such as depigmentation and tendon rupture [34-36]. Our study continues to build up on the rising evidence of the safety and efficacy of PRP in tendinopathy; while the use of PRP in De Quervain’s tendinopathy is not as common as some of the aforementioned tendinopathies, the results from this systematic review justify the need for further studies that assess the potential of PRP in treatment of De Quervain’s tendinopathy.

This systematic review aimed to evaluate the efficacy of PRP use in the treatment of DQVD. Assessment of the literature showed several studies of varying quality in support of the efficacy of PRP in DQVD. The studies assessed showed a significant reduction in VAS and DASH scores with the use of PRP across various time points and various settings. The assessment also revealed the efficacy of PRP when compared to conventional steroid injections as well as when compared to standard conservative management. Additionally, the safety profile of PRP across all studies assessed was excellent with a very small number of transient injection-related complications.

While our systematic review yielded good evidence favoring the use of PRP in De Quervain’s tendinopathy, we have also identified significant areas that need structured scrutiny to achieve uniform support for PRP use. Studies in this systematic review have used very wide variations of PRP preparation technique which was been shown to significantly alter outcomes in the treatment of tendinopathy with highly cellular preparation techniques showing a more positive outcome [37]. Future studies should aim to adopt a unified PRP preparation technique with a focus on using techniques that yield a highly cellular formulation of PRP.

The lack of uniformity in studies is further complicated by some studies injecting the PRP using a landmark technique and other studies using ultrasound guidance to aid in the accurate injection of PRP into the desired area. Studies have shown that ultrasound guidance is superior in the accuracy of injection when compared to landmark-guided injections, especially in the hands of less experienced providers [47,48]. Unification of the administration method is paramount to the determination of the efficacy of the injection administered and further studies should aim to use the guidance of ultrasound to aid injection of PRP into the peritendinous area. This consideration is of greater importance in settings where providers have varying levels of experience in administering peritendinous injections.

This systematic review has identified several studies assessing the use of PRP in the treatment of De Quervain’s tenosynovitis. Most of the studies were experimental comparative studies with three recent medium-quality randomized controlled trials. In the literature reviewed, PRP appears to be a safe and effective treatment modality for De Quervain’s tenosynovitis. PRP has demonstrated improvements in DASH, VAS, PRWE, and Mayo wrist scores across the studies reviewed across various time points and despite various preparation and administration methods. The studies that used ultrasound for assessment of tendon thickness also showed significant efficacy of PRP in reducing the inflammatory response of the tendon sheath. While most of the studies demonstrated the efficacy of PRP and its superiority to conventional steroid injections, this finding was not as uniform, and some studies have shown comparable effects to steroids with no significant difference in efficacy across various time points.

The conclusions that are asserted by this study are limited by the paucity of high-quality studies. Studies included in this review used a variety of different assessment scores to measure the therapeutic effect of PRP, which greatly limits the comparability and verification of results across studies. VAS score was the most utilized scoring system across the studies due to its relatively simple nature. Other scores, like DASH score, Mayo wrist score, and PRWE score, are more detailed and provide more information on an individual basis but were less generally adopted across all studies. Future studies should aim to adopt at least the VAS, DASH score, and one of the Mayo wrist or PRWE scores to facilitate comparison with established literature. Meaningful meta-analysis was not possible due to the small number of RCTs, significant heterogeneity of the studies, and lack of standardization of intervention, comparison groups, and follow-up duration.

This review showed that there is an increasing amount of compelling evidence to support the use of PRP in De Quervain’s tenosynovitis. This is in line with emerging evidence of the efficacy of PRP use in various tendinopathies. There is, however, insufficient evidence currently to support the superiority of PRP to conventional steroid injections. Further high-quality randomized studies are needed to further clarify the effectiveness of PRP in the treatment of De Quervain’s tenosynovitis. Studies aiming to discern the efficacy of PRP should adopt a unified preparation and administration technique and compare its use with both conservative therapy and steroid injections.

Limitations

The studies included in this systematic review showed several limitations. Significant variability is apparent between all studies in various aspects from study designs, methodology, PRP preparation, and administration, and extends to variability in scoring and follow-up methodology.

Preparation and administration of PRP were significantly variable between studies, with major differences in centrifugation speed and time. Moreover, injected PRP amount was highly variable between studies as well. Injection administration was also not uniform with two studies opting for ultrasound-guided injection while the rest injected the PRP using anatomic landmarks. This could greatly affect the results of each of the studies and is a limitation of interpretation of the results.

Not all studies included a control group or a comparison group. While five studies included a comparison group against corticosteroids, only two studies included a control group in the form of conservative management. One of the studies included in the analysis had no control or comparison group. None of the studies included a placebo control group which may help in improving the certainty of improvement attributed to PRP or steroid injection.

Confounding interventions were also noted in some of the studies with one of the studies including surgical intervention [20] and another one [30] including a concomitant tenotomy at the time of PRP injection. The sample size was uniformly small in all the studies. This limitation was more pronounced in the study conducted by Assad et al., which only included 12 subjects. This may affect the generalizability of findings in these studies to a larger population.

## Conclusions

Studies in this review have presented PRP as a possible practical and affordable modality for the treatment of De Quervain tenosynovitis. Most of the studies in the review have demonstrated improvement that is comparable to steroid injections with a very limited side effect profile. In conclusion, PRP is a promising and safe alternative to conventional steroid injections in the treatment of De Quervain’s tenosynovitis. Further large sample studies are needed prior to the definitive recommendation of PRP as the gold standard for the treatment of De Quervain’s tenosynovitis.
